# Changing Patterns of SARS-CoV-2 Seroprevalence among Canadian Blood Donors during the Vaccine Era

**DOI:** 10.1128/spectrum.00339-22

**Published:** 2022-04-12

**Authors:** Cassandra N. Reedman, Steven J. Drews, Qi-Long Yi, Chantale Pambrun, Sheila F. O’Brien

**Affiliations:** a Epidemiology and Surveillance, Canadian Blood Services, Ottawa, Ontario, Canada; b Medical Microbiology Department, Canadian Blood Services Edmonton, Edmonton, Alberta, Canada; c Department of Laboratory Medicine & Pathology, Division of Diagnostic and Applied Microbiology, University of Alberta, Edmonton, Alberta, Canada; d School of Epidemiology and Public Health, University of Ottawa, Ottawa, Ontario, Canada; e Medical Affairs & Innovation, Canadian Blood Services, Ottawa, Ontario, Canada; f Department of Pathology & Laboratory Medicine, University of Ottawa, Ottawa, Ontario, Canada; Quest Diagnostics

**Keywords:** SARS-CoV-2, seroprevalence, Canada, blood donors

## Abstract

We monitored the seroprevalence of severe acute respiratory syndrome coronavirus 2 (SARS-CoV-2) nucleocapsid (anti-N; proxy of natural infection) and spike protein (anti-S; proxy for humoral immunity) antibodies in blood donors across Canada from January to November 2021. The first and second doses of vaccine were deployed over this time. Anti-N seroprevalence remained low overall (about 5% or lower) from January to November but was higher in racialized groups, younger age groups, and those living in materially deprived neighborhoods. Anti-S seroprevalence corresponded with the roll out of vaccines across the country, increasing in April in older donors and then progressively to younger age groups consistent with vaccination policies targeting oldest to youngest. By November, close to 100% of blood donors were positive for anti-S. Anti-S concentrations peaked by July and began waning by September to November particularly in older donors. These data have informed national and provincial public health policy in Canada throughout vaccination rollout.

**IMPORTANCE** Throughout the severe acute respiratory syndrome coronavirus 2 (SARS-CoV-2) pandemic, our blood donor seroprevalence study has informed Canadian public health policy at national and provincial levels. We describe the only continuously running national seroprevalence study in Canada, which spans the full length of the pandemic and per capita is one of the largest programs in the world. The benefit of seroprevalence studies is that they identify a broad range of asymptomatic and symptomatic infection histories that may not be identified with active SARS-CoV-2 nucleic acid testing programs or when case definitions change. As vaccination was deployed in Canada, we estimated the proportion of donors with vaccine-related antibodies and developed population-level estimates of SARS-CoV-2 spike antibody concentrations. Monthly predictive mathematical models and our results engaged public health organizations in new ways. In the future, we intend to continue to expand on these interactions with provincial and national public health teams.

## INTRODUCTION

In Canada, approximately 1.8 million severe acute respiratory syndrome coronavirus 2 (SARS-CoV-2) cases were reported as of 24 November 2021 (1). Early in the pandemic, strict public health policies (with some variability by region) reduced the spread of SARS-CoV-2 (2). The first wave of coronavirus infection disease 2019 (COVID-19) in Canada subsided by July 2020 and plateaued over the summer. A second wave followed in September 2020, with a resurgence in March and April 2021. A fourth wave that began in early August 2021 was subsiding by November.

In April 2020, the Canadian Federal Government formed the COVID-19 Immunity Task Force to coordinate research and inform policy addressing the pandemic (https://www.covid19immunitytaskforce.ca/). Canadian Blood Services (CBS) was tasked with monitoring SARS-CoV-2 seroprevalence starting in May 2020 with over 250,000 blood samples tested to date. Routine COVID-19 case reports and testing underestimate the total number of infections because individuals with mild symptoms or asymptomatic infections may not be identified or seek testing (3, 4). Seroprevalence studies estimate the population prevalence of individuals with SARS-CoV-2-specific antibodies independent of symptoms (5–8) and monitor vaccine-related antibodies.

Blood donor seroprevalence studies have informed SARS-CoV-2 public health policies around the world (9). Although not fully representative of the general population, blood donors are a large healthy population, permitting cost-effective and efficient wide-scale evaluation of SARS-CoV-2 seroprevalence and trends across demographic groups (5, 8, 10). With blood collections in 9 of 10 provinces in all larger cities and most smaller urban areas, our study has near national coverage and is the only Canadian serosurvey with monthly sampling over the duration of the pandemic. To date we have published one report from our study that described seroprevalence in the first wave of the pandemic (7). Other Canadian serosurveys have been carried out over limited time periods and relied on blood spots for serology (11, 12) or have been limited to specific regions (13, 14) and/or populations (15–17). In other countries, such as the United States, blood donors are central to national monitoring of infections and vaccine seroprevalence (5, 18) but have not published data on vaccine-related antibody concentrations. Interpretation of waning postvaccination antibody concentrations in relation to immunity is unclear but may correlate with increased infection risk (19–21); therefore, monitoring will refine mathematical models estimating timing of subsequent vaccine doses.

Of 5 vaccines approved by Health Canada, only 4 have been administered. The most widely used was Pfizer-BioNTech Comirnaty, followed by Moderna Spikevax, AstraZeneca Vaxzevria (AstraZeneca), and COVISHIELD (Serum Institute of India) (22). Vaccines became available to certain groups of individuals (immunocompromised, elderly population, etc.) in Canada beginning in late December 2020/January 2021. Vaccine eligibility in Canada gradually extended to age groups starting with those over 80 years of age and opened to most adults by spring 2021. The aim of this study is to monitor the seroprevalence of spike (anti-S) and nucleocapsid (anti-N) antibodies in Canadian blood donors aged 17 and older over the 11 months of SARS-CoV-2 vaccine rollout.

## RESULTS

**FIG 1:**
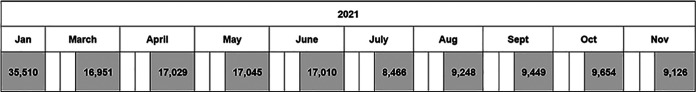
Number of retention samples tested by month from January to November 2021. A larger sample was tested in January, however, in February, samples were not tested. From March onward, samples from approximately the last 2 weeks of every month were tested; Jan, January; Aug, August; Sept, September; Oct, October; Nov, November.

Between 1 January and 24 November 2021, retention samples from 149,522 donations were screened for SARS-CoV-2 anti-S and anti-N (Fig. 1). Table 1 shows blood donor demographics compared with the Canadian population. Compared with the general population, the proportion of male donors was slightly higher, and there was some variability by regions, but age group and ethnicity were similar. Applying weighting for population and adjustments for test characteristics did not change seroprevalence estimates substantially for any demographic group.

**TABLE 1:** Study demographics compared to Canadian population demographics

Item	Categories	CBS data	Canadian population^*a*^
		%	Rate (%)
Sex	Female	42.2%	50.3%
	Male	57.8%	49.7%
Age group	17–29	18.3%	21.7%
	30–39	18.0%	19.0%
	40–59	35.9%	35.5%
	60–69	20.7%	17.2%
	70+	7.1%	6.6%^*b*^
Ethnicity^*c*^^,^^*d*^	White	75.0%	77.7%
	Racialized	15.4%	22.3%
Region	British Columbia	16.4%	17.7%
	Alberta	20.9%	15.1%
	Prairies	11.1%	8.7%
	Ontario	40.6%	50.2%
	Atlantic Canada	11.0%	8.4%

a Statistics Canada available from https://www150.statcan.gc.ca/n1//en/type/data?MM=1#tables.

b Ages 70 to 75 in Canada.

c Statistics Canada Census (2016) available from https://www12.statcan.gc.ca/census-recensement/2016/dp-pd/prof/index.cfm?Lang=E.

d Some missing data for all provinces.

**FIG 2:**
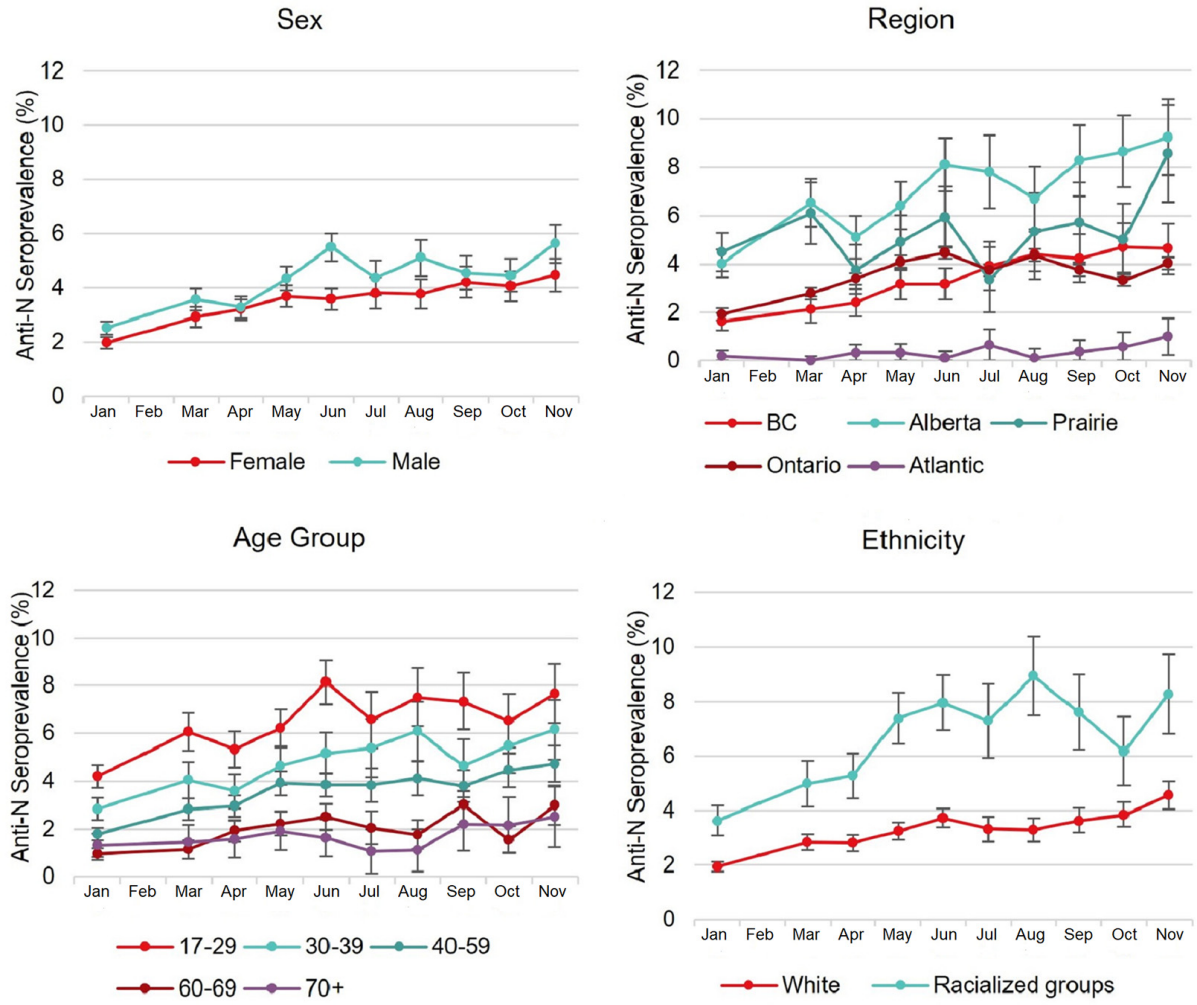
Temporal trends of nucleocapsid antibody seroprevalence (with 95% confidence intervals) from January to November 2021 by sex, age group, ethnicity, and region.

**FIG 3:**
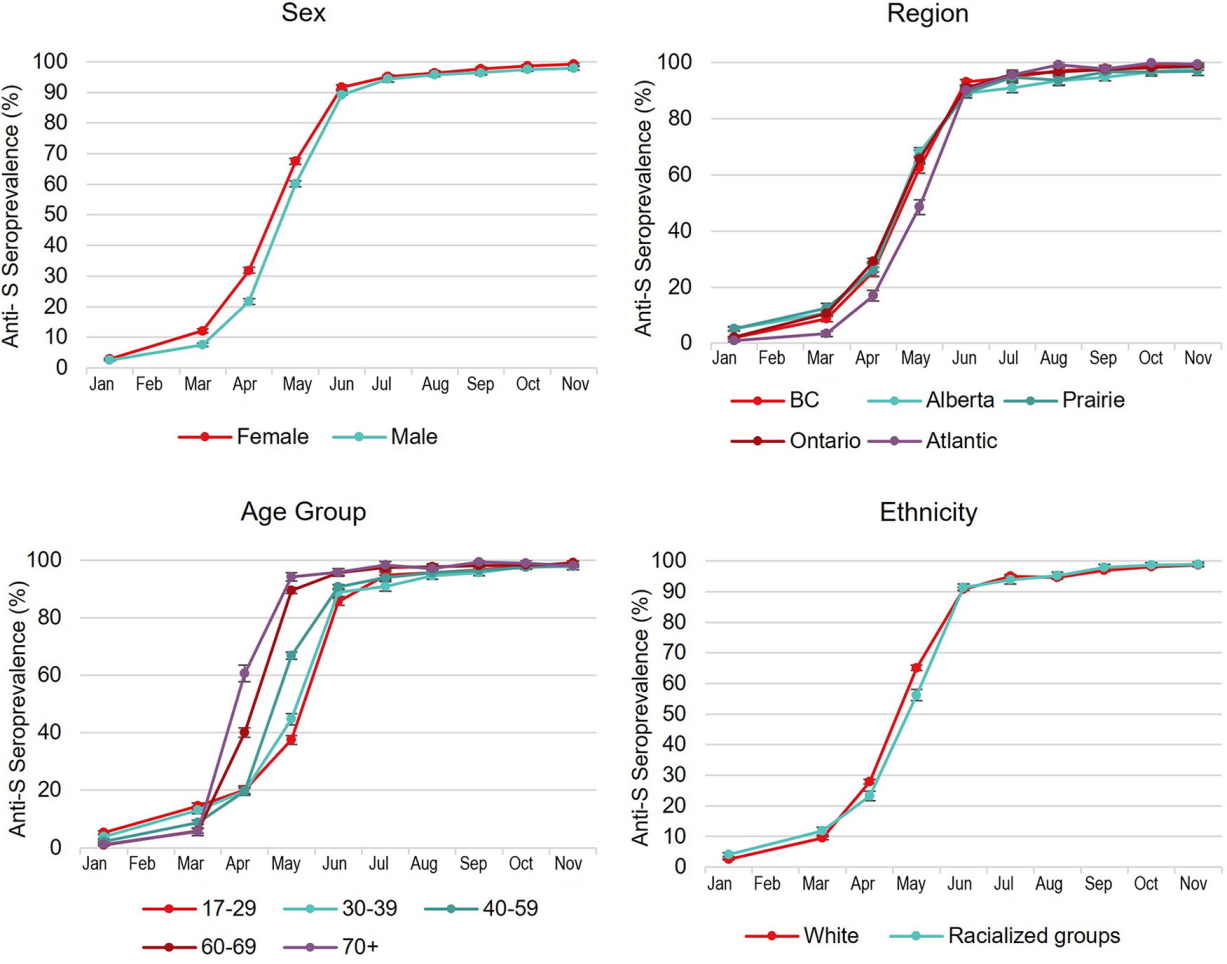
Temporal trends of spike antibody seroprevalence (with 95% confidence intervals) from January to November 2021 by sex, age group, ethnicity, and region.

The anti-N seroprevalence ranged from 2.24% (95% confidence interval [95% CI] 2.08, 2.41%) in January to 5.04% (95% CI 4.58, 5.50%) in November, and anti-S ranged from 2.80% (95% CI 2.60, 3.00%) in January to 98.52 (95% CI 98.18, 98.86%) in November. Figure 2 and 3 illustrate temporal trends of anti-N and anti-S seroprevalence by demographic groups (sex, age, ethnicity, and region) at monthly intervals. In univariate models, a higher proportion of anti-N positivity was associated with racialized groups, younger age, less social deprivation, and living in neighborhoods with greater material deprivation (all comparisons *P < *0.05). When the anti-N univariate models were refitted with interaction terms, significant interactions (*P < *0.05) were detected between month and ethnicity in May; month and age group in May, June, and October; month and material deprivation in March, June, July, September, and October; and month and social deprivation in March, May to August, and November.

In univariate models, a higher proportion of anti-S positivity was associated with older age, being female, living in a more affluent neighborhood, and less social deprivation (all comparisons *P < *0.05). Significant interactions (*P < *0.05) were detected between month and ethnicity in April to July; month and age group in March to November; month and sex in March, May, June, and August; month and material deprivation in April to October; and month and social deprivation in March to June, August, and October. Demographic variables (ethnicity, sex, age group, Pampalon Material and Social Deprivation Indices [MSDI], region, and month) were independent predictors of both anti-N and anti-S positivity in multivariable logistic regression models (Tables S1 and S2 in the supplemental material). Racialized groups, being male, younger age, higher material deprivation, less social deprivation, living in Ontario, Alberta, or the Prairies, and later month were predictors of higher anti-N seropositivity (Table S1). Racialized groups, being female, older age, less material deprivation, less social deprivation, living in Ontario, and later month were predictors of higher anti-S seropositivity (Table S2).

**FIG 4:**
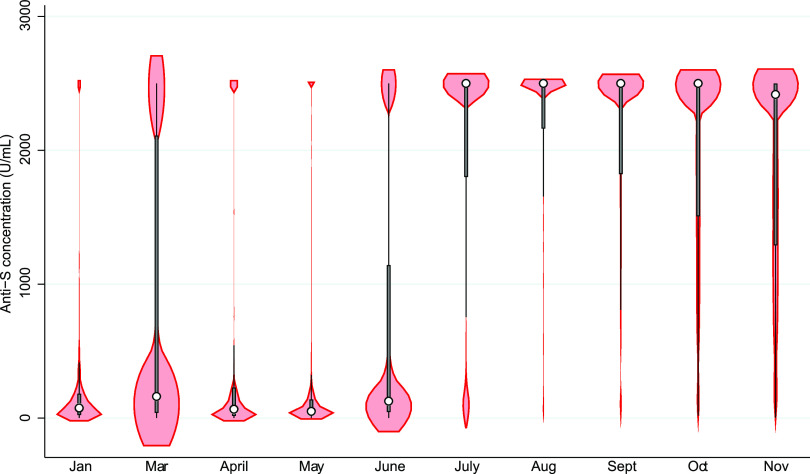
Overall temporal trends and distributions of spike antibody concentration (U/mL) by month from January to November 2021 (all values >2,500 U/mL classified as 2,500 U/mL; the white circle represents the median, and the thick bar represents the interquartile range [IQR]).

**FIG 5:**
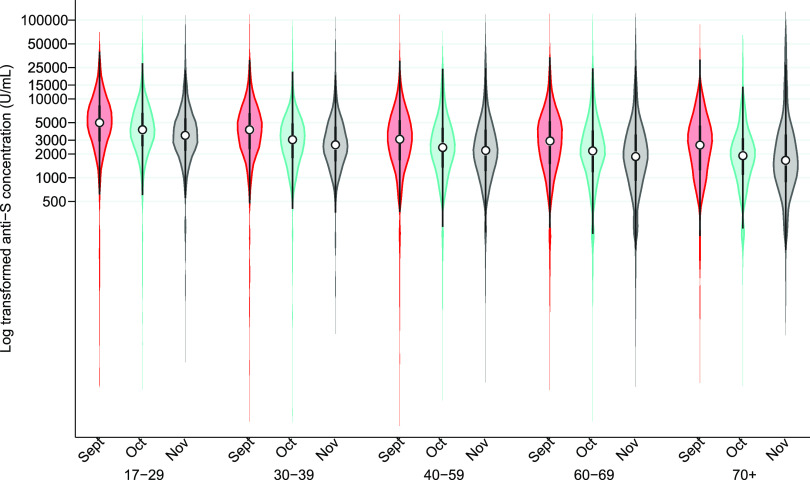
Distributions of spike antibody concentration (U/mL) by age group in spike antibody-positive donations in September (red), October (blue), and November (maroon) (dilution of 1:400 allows detection of up to 100,000 U/mL; the white circle represents the median, and the thick bar represents the interquartile range [IQR]).

Figure 4 shows the kernel density plot of the distribution and median anti-S concentration by month in all anti-S-positive donations. The limit of detection was 2,500 arbitrary units/mL (U/mL); September, October, and November values equal to or greater than 2,500 U/mL were set at 2,500 to be comparable with previous months in Fig. 4. Median concentrations of anti-S remained similar from January (76.8 U/mL, interquartile range [IQR] of 158.2 U/mL) to May (50.0 U/mL, IQR of 122.9 U/mL) with an increase in June (126.0 U/mL, IQR of 1,099.8 U/mL) and a much larger increase in July (2,500 U/mL, IQR of 694 U/mL). The piecewise linear regression model identified a significant interaction (*P < *0.001) between breakpoint (July) and age group. This age association is also observed in Fig. 5 (using a 1:400 dilution method and log transformation). In a linear regression model of September, October, and November data with anti-S concentrations as the dependent variable, there was a negative slope with older age group (*P < *0.001) and later month (*P < *0.001). The median concentration (all age groups combined) in September was 3,471 U/mL (IQR of 4,231 U/mL), in October it was 2,725 U/mL (IQR of 3,258 U/mL), and in November it was 2,416 U/mL (IQR of 3,086 U/mL).

## DISCUSSION

This is the only study in Canada to evaluate SARS-CoV-2 antibody seroprevalence and concentrations coast to coast on a continuous basis over the full deployment of first and second doses of vaccines. The proportion of anti-N-positive samples (natural infection) was elevated in racialized groups and younger age groups and among those living in materially deprived neighborhoods. Regional variation was also observed, with higher proportions of anti-N-positive samples in the western provinces and very low proportions in the Atlantic region. The proportion of samples positive for anti-S had largely peaked by June 2021, increasing first in the oldest individuals and progressively the younger age groups. Antibody concentrations reached very high levels by July with waning by September to November, particularly in older age groups. These data are an integral part of Canada’s SARS-CoV-2 surveillance and are made available to provincial and federal public health departments and mathematical modelers to inform public health policy.

### Natural infection.

In Canada, the seroprevalence for natural infection remained below or around 5% over the study period. This is similar to pooled seroprevalence estimates in some countries, including Spain, England, France, and Norway (23, 24), but lower than the 20% reported in U.S. blood donors by May 2021 (5). This mirrors reported case data with 4,539 per 100,000 population in Canada (1), 5,000 in Spain (24), 5,549 in England (24), 2,504 in France (24), and 3,190 in Norway (23) but 14,089 per 100,000 population in the United States (25). Higher prevalence of infection in racialized groups and those from neighborhoods with lower socioeconomic status in our study is consistent with higher incidence of COVID-19 morbidity and mortality in these groups in Canada and other countries (5, 26–29), thought to be related to higher density living and employment with more social contacts.

Regional differences were detected in our data, which may reflect variability in policies responding to the pandemic. Consistent with public health case data, we observed higher natural infection rates in Alberta and the Prairie region and lowest rates in Atlantic Canada. While there was a steady increase observed in our data across almost all regions from January to November, the natural infection rate in the Prairie region increased more substantially from October to November. We also observed that younger people had higher anti-N seropositivity. This is consistent with reported cases in Canada (1). This pattern was evident as early as January, prior to widespread vaccination, likely related to social behaviors. With greater risk of severe symptoms with increasing age, older individuals were more likely to follow public health guidelines, hence lower seroprevalence and lower proportions of reported cases in the older population (1). Seroprevalence in other countries such as the United States follows a similar pattern (5, 18).

### Vaccination.

Anti-S seroprevalence of essentially all donors in our study shows very high vaccine uptake. This is higher than the 86% of individuals 12 and older in the general population fully vaccinated as of 20 November 2021 (22). Blood donors may be more health conscious and less vaccine hesitant than some nondonors. While not fully representative of the general population, the high vaccination rates in donors are ideal for monitoring waning anti-S concentrations and breakthrough infections. We note that some S positivity may be due to natural infection with or without vaccination, but with about 5% anti-N positive, most are vaccination related. Our study exposed inequities in certain communities, such as racialized groups and individuals living in materially deprived neighborhoods. While the greater infection risks in these groups have been noted by others as previously mentioned, SARS-CoV-2 vaccine inequity has largely flown under the radar in Canada, with only a few reports on local communities (29).

In Canada, vaccination prioritization varied by region but tended to focus on high-risk groups initially, such as long-term care residents and health care workers. As vaccine availability increased by March/April 2021, older Canadians were prioritized then gradually expanded to younger people (22). Anti-S seroprevalence mirrors these policies, with increases observed temporally from the oldest to the youngest.

An important feature of our program is monitoring of anti-S concentration. While the proportion of donors with anti-S increased progressively over the period, the concentration of antibody remained relatively low until July. In Canada, due to limited supply, many jurisdictions delayed the second dose until 4 months after the first (30) in order to provide a first dose to as many people as possible. By late June/early July, the second dose was being widely administered. Because the maximum concentration that could be detected up until August was 2,500 U/mL, it is unclear how high the median concentration was in July and August. By changing the dilution of the samples, we were able to measure concentrations as high as 100,000 U/mL from September onward. Concentrations remained very high, but there was a clear trend toward lower concentration with increasing age. As receipt of the second dose was prioritized from oldest to youngest, this likely reflects a waning antibody response, although a muted antibody response in older individuals could contribute (19, 31, 32). Reports of antibody concentrations in focused kinetics studies suggest that antibody concentrations peak after about 1 to 3 months and gradually decrease thereafter (19, 33). There are very few population studies tracking postvaccination anti-S concentrations, and the application as a correlate of immunity has yet to be elucidated. In a study of about 5,000 health care workers in one hospital in Israel, steady reduction in antibody concentration was reported over 6 months postvaccination, but neutralizing titers dropped for the first 3 months, after which they remained stable (19). In tested-only design studies, vaccine efficacy was highest in the first month postvaccination and decreased by 3 to 4 months (21, 34) but largely prevented hospital admissions over 6 months of tracking (34). At an individual level, the predictive value of antibody concentration for infection risk is uncertain. However, at a population level, it is anticipated that concentration data generated on an ongoing basis in our study will be integral to mathematical models to inform roll out of further vaccine doses. Further research is needed to refine such modeling.

### Limitations.

Our study has limitations. Data on vaccination or infection history were not available. Data on specific variants of concern (VOCs) were also unavailable. Beginning in January 2021, the dominant VOC was Alpha (B.1.1.7), which remained the dominant VOC in the population until late June 2021 when Delta (B.1.617.2) became dominant and remained so for the duration of the study (1). Anti-N may wane faster than anti-S after an infection has subsided (35, 36), thus the percentage of anti-N positivity underestimates cumulative infection (5, 37). COVID-19-based deferrals (i.e., recent exposure/contact with an infected individual or symptomatic-based deferral) were rare (<0.1%) but more may have delayed donation, which could create a lag in measurement of anti-N infection rate. Although we weighted the data for general population age and sex by region, this may not fully adjust our estimates to represent the general population of Canada. Blood donors are healthy people aged 17 and older; therefore, we are not capturing data from many important subpopulations within the country, including long-term care residents, prison inmates, children, and those who do not meet blood donor criteria.

### Conclusion.

We have monitored SARS-CoV-2 seroprevalence over 11 months of vaccine rollout in a Canada-wide study of blood donors. Infection-related seroprevalence remained low but was elevated in racialized groups, people living in materially deprived neighborhoods, and younger people. The proportion of people with vaccination-related antibodies increased in the oldest donors first, consistent with age prioritization vaccination policies. By July 2021, vaccination-related antibody concentrations were very high, consistent with timing of the second dose, but thereafter decreased. As the SARS-CoV-2 global pandemic progresses and vaccine efficacy/duration is better understood, continuous seroprevalence studies will be vital for determining the use or timing of potential future boosters and will be an important national monitoring system as new variants arise.

## MATERIALS AND METHODS

### Study design and population.

Donors must be a minimum of 17 years of age to donate their blood. Prior to donating blood, donors are required to answer screening questions to ensure that they are in good health and are not at risk of blood-transmissible infections. To reduce the risk of SARS-CoV-2 for donors and staff at the collection site, all donors are deferred for 2 weeks from donating blood if they have been in contact with someone who was infected or if they have had an infection (3 weeks if hospitalized). Donors also have their temperature checked to ensure that they are afebrile before donating. These deferrals remained constant for the entirety of sample collection for this study. Canadian Blood Services collects donations from all provinces (excluding Quebec); however, no samples are collected in the territories. Depending on sex and donation type, donors were able to donate multiple times within this study period and had a chance of being selected for the study; therefore, there were some repeat donors that had more than one sample tested. However, because both spike and nucleocapsid seropositivity status could change over the course of the study, and because each month was considered a snapshot of seroprevalence, these samples were included. An extra EDTA blood sample is collected from all donors at the time of donation; approximately 80% of these samples are not required for qualification of the blood products and were therefore available for serological testing in this study. These residual samples were collected from approximately the last 2 weeks of every month from January 2021 to November 2021, as shown in Fig. 1. A straight random sample was applied up until June, after which samples were stratified into age groups by region before being randomly selected to reduce the total monthly sample size while maintaining a sample representative of important population demographics (age and region). This study was approved by the Canadian Blood Services Research Ethics Board.

### Serologic testing.

Retention EDTA plasma samples were aliquoted and frozen at −20°C or colder until the time of testing and were processed at the Canadian Blood Services laboratory in Ottawa, ON. All samples were tested using two assays: the Roche Elecsys anti-SARS-CoV-2 spike semiquantitative immunoassay (Roche Diagnostics International Ltd., Rotkreuz, Switzerland), which measures total antibodies (including IgA, IgM, and IgG) to the SARS-CoV-2 spike protein (anti-S), and the Roche Elecsys anti-SARS-CoV-2 qualitative immunoassay (Roche Diagnostics International Ltd., Rotkreuz, Switzerland), which measures total antibodies (including IgA, IgM, and IgG) to SARS-CoV-2 recombinant protein nucleocapsid antigen (anti-N). Samples from January to August were tested neat for anti-S and also at a 1:10 dilution if above the maximum level of detection; however, by July, many samples were above the maximum detection level when diluted. Beginning in September, the dilution was increased to 1:400. Evaluation of 100 samples that tested greater than 250 U/mL neat but less than 2,500 at a 1:400 dilution also tested at a 1:10 dilution was strongly correlated (0.977, *P < *0.001). At a concentration of ≥0.8 U/mL, the anti-S assay was assumed to have a sensitivity of 98.8% and specificity of 99.97% (38). At a sample-to-cutoff ratio of ≥ 1.0, the anti-N assay was assumed to have a sensitivity of 99.5% and specificity of 99.8% (39).

Serological testing using the anti-N and anti-S assays allows trends in natural infection and vaccine-induced seropositivity to be monitored (5). The primary outcomes were the infection-induced SARS-CoV-2 seroprevalence (defined as samples that tested positive for anti-N, also referred to as natural infection [samples that also tested anti-S seropositive were not excluded from this analysis]), primarily vaccine-induced SARS-CoV-2 seroprevalence (defined as samples that tested positive for anti-S, also referred to as humoral immunity [samples that also tested anti-N positive were not excluded from this analysis]), and anti-S concentration (measured in arbitrary units/mL [U/mL]).

### Data management and statistical analysis.

Demographic variables were extracted from the Canadian Blood Services donor database and added to the test data, including donation date, forward sortation area (FSA) from the residential postal code, sex, age, and self-reported ethnicity. Provinces were classified into geographical regions across Canada (British Columbia, Alberta, Prairies [included Saskatchewan and Manitoba], Ontario, and the Atlantic region [included New Brunswick, Nova Scotia, Prince Edward Island, and Newfoundland and Labrador]). Donors self-identified as white, Asian, Indigenous, Arabic, black, South Asian, Latin American, or other. Ethnicities were regrouped *a priori* as either “white” (the majority of donors) or “racialized groups” because the proportions in various nonwhite ethnicities were small. Socioeconomic status was estimated by the Pampalon Material and Social Deprivation Indices (MSDI) (40, 41). Material deprivation is associated with insecure job situation, insufficient income, and low education, while social deprivation refers to a fragile social network, characterized by living alone, being a single parent, or being separated, divorced, or widowed. MSDI was derived from the 2016 Statistics Canada census, aggregated from postal codes to the dissemination area (DA) level (the smallest geographic unit available in the Canadian census, considering 400 to 700 persons), and were categorized into quintiles from least deprived (1) to most deprived (5). Donors were categorized into 5 different age groups: 17 to 29, 30 to 39, 40 to 59, 60 to 69, and 70+ years old. Data were weighted by raking for the donor’s forward sortation area (FSA), age group, and sex to make inference to the general population based on Statistics Canada data (catalog number 98-400-X2016008). For FSAs with few donors, several adjacent FSAs were combined to include at least 500 donors. In cases where no FSA was recorded or if not in a province where blood is collected (0.2% of samples), weighting was based on FSA of the blood center.

The weighted data were adjusted for sensitivity and specificity of the assay using the Rogan-Gladen equation (42). The seroprevalence was calculated as the number of positive samples divided by all samples tested, and the exact method was used to estimate 95% confidence intervals (95% CIs). SARS-CoV-2 seroprevalence was stratified by region, sex, age group, self-reported ethnicity, and MSDI by month.

Associations and risk factors for vaccine-induced humoral immunity (anti-S positive) and natural infection (anti-N positive) by month were evaluated using logistic regression. Univariate and multivariate logistic regression were used to compare demographics of anti-S- and anti-N-positive donors to nonpositive donors, resulting in two multivariable logistic regression models (one for anti-S and one for anti-N). Independence of variables was evaluated in the multivariable models. Interactions were tested in univariate models between each demographic variable and month (modeled as a categorical variable).

Anti-S concentration by month was evaluated through linear regression. A piecewise linear regression model was fitted in which the slope was permitted to vary at the approximate date when concentrations began decreasing rather than increasing (breakpoint), with before and after breakpoint, sex, and age group as predictors. Interaction between breakpoint and age group was included in the model to assess the relationship between age and waning anti-S concentration. A separate linear regression model was used to examine the relationship between age group and anti-S concentration from September to November (when a 1:400 dilution allowed up to 100,000 U/mL detection).

All analyses were conducted using SAS (version 9.4, Cary, NC) and STATA/MP 17 (Statacorp. 2021, College Station, TX). Data may be made available upon request from Canadian Blood Services (contact S.F.O.) subject to internal review, privacy legislation, data sharing agreements, and research ethics approval.
