# Supplementary material for: Changing Patterns of SARS-CoV-2 Seroprevalence among Canadian Blood Donors during the Vaccine Era
Source: Microbiol Spectr. 2022 Apr 12;10(2):e00339-22. doi: 10.1128/spectrum.00339-22 (PMC9045380; doi:10.1128/spectrum.00339-22)
Supplement: SUPPLEMENTAL FILE 1 — Supplemental material. Download spectrum.00339-22-s0001.pdf, PDF file, 0.1 MB [file spectrum.00339-22-s0001.pdf]

Table A1. Results from multivariable logistic regression model (outcome = Nucleocapsid antibody seropositive) from blood donors across Canada between January and November 2021.

Variables	Odds Ratio (OR)	*P*-value	95% CI
**Ethnicity**			
White	Ref.	-	-
Racialized Groups	1.45	< 0.001	1.35 – 1.56
**Sex**			
Female	Ref.	-	-
Male	1.11	0.002	1.04 – 1.18
**Age Group**			
17 – 29	Ref.	-	-
30 – 39	0.73	< 0.001	0.67 – 0.79
40 – 59	0.60	< 0.001	0.56 – 0.65
60 – 69	0.37	< 0.001	0.34 – 0.41
70+	0.37	< 0.001	0.32 – 0.44
**Material Deprivation**			
Quintile 1[a]	Ref.	-	-
Quintile 2	1.19	< 0.001	1.09 – 1.30
Quintile 3	1.30	< 0.001	1.18 – 1.42
Quintile 4	1.75	< 0.001	1.59 – 1.92
Quintile 5[b]	1.90	< 0.001	1.70 – 2.12
**Social Deprivation**			
Quintile 1[a]	Ref.	-	-
Quintile 2	0.88	0.005	0.80 – 0.96
Quintile 3	0.78	< 0.001	0.71 – 0.86
Quintile 4	0.73	< 0.001	0.67 – 0.80
Quintile 5[b]	0.74	< 0.001	0.68 – 0.82
**Region**			
British Columbia	0.96	0.38	0.87 – 1.05
Alberta	2.00	< 0.001	1.86 – 2.15
Prairies	1.37	< 0.001	1.24 – 1.51
Ontario	Ref.	-	-
Atlantic Canada	0.14	< 0.001	0.11 – 0.17
**Month**			
January	Ref.	-	-
March	1.36	< 0.001	1.20 – 1.54
April	1.44	< 0.001	1.28 – 1.64
May	1.80	< 0.001	1.61 – 2.02
June	2.04	< 0.001	1.82 – 2.28
July	1.91	< 0.001	1.66 – 2.20
August	1.83	< 0.001	1.59 – 2.10
September	2.04	< 0.001	1.79 – 2.34
October	2.05	< 0.001	1.80 – 2.34
November	2.50	< 0.001	2.20 – 2.84

*Note: Ref. = Referent in the model

[a] Least deprived

[b] Most deprived

Table A2. Results from multivariable logistic regression model (outcome = Spike antibody seropositive) from blood donors across Canada between January and November 2021.

Variables	Odds Ratio (OR)	*P*-value	95% CI
**Ethnicity**			
White	Ref.	-	-
Racialized Groups	1.15	< 0.001	1.09 – 1.22
**Sex**			
Female	Ref.	-	-
Male	0.71	< 0.001	0.68 – 0.74
**Age Group**			
17 – 29	Ref.	-	-
30 – 39	1.01	0.76	0.95 – 1.08
40 – 59	1.36	< 0.001	1.29 – 1.44
60 – 69	2.59	< 0.001	2.43 – 2.76
70+	3.72	< 0.001	3.40 – 4.07
**Material Deprivation**			
Quintile 1[a]	Ref.	-	-
Quintile 2	0.86	< 0.001	0.82 – 0.91
Quintile 3	0.81	< 0.001	0.77 – 0.86
Quintile 4	0.75	< 0.001	0.70 – 0.79
Quintile 5[b]	0.82	< 0.001	0.76 – 0.88
**Social Deprivation**			
Quintile 1[a]	Ref.	-	-
Quintile 2	0.95	0.08	0.89 – 1.01
Quintile 3	0.86	< 0.001	0.81 – 0.92
Quintile 4	0.92	0.006	0.86 – 0.97
Quintile 5[b]	0.87	< 0.001	0.82 – 0.92
**Region**			
British Columbia	0.92	0.007	0.87 – 0.98
Alberta	1.01	0.60	0.96 – 1.07
Prairies	1.00	0.89	0.94 – 1.08
Ontario	Ref.	-	-
Atlantic Canada	0.64	< 0.001	0.60 – 0.68
**Month**			
January	Ref.	-	-
March	3.34	< 0.001	3.05 – 3.66
April	11.99	< 0.001	11.08 – 12.97
May	61.53	< 0.001	56.90 – 66.53
June	344.67	< 0.001	314.97 – 377.16
July	565.79	< 0.001	502.08 – 637.60
August	757.40	< 0.001	667.12 – 859.91
September	901.13	< 0.001	789.24 – 1028.89
October	1311.22	< 0.001	1129.02 – 1522.82

November	3224.55	< 0.001	2587.77 – 4018.02

*Note: Ref. = Referent in the model

[a] Least deprived

[b] Most deprived
